# Effect of Sheet Vibration on the Theoretical Analysis and Experimentation of Nonwoven Fabric Sheet with Back Air Space

**DOI:** 10.3390/ma15113840

**Published:** 2022-05-27

**Authors:** Shuichi Sakamoto, Ryo Iizuka, Takumi Nozawa

**Affiliations:** 1 Department of Engineering, Niigata University, Ikarashi 2-no-cho 8050, Nishi-ku, Niigata City 950-2181, Japan; 2 Graduate School of Science and Technology, Niigata University, Ikarashi 2-no-cho 8050, Nishi-ku, Niigata City 950-2181, Japan; t16m007d@gmail.com (R.I.); t18a093a@gmail.com (T.N.)

**Keywords:** nonwoven fabric sheet, back air space, vibration, absorption coefficient

## Abstract

The purpose of this study was to improve the accuracy of the theoretical analysis of sound absorption mechanisms when a back air space is used in nonwoven fabrics. In the case of a nonwoven sheet with a back air space, it can be shown that there is a difference between the experimental results and theoretical analysis results obtained using the Miki model when the area of the nonwoven sheet is large. Therefore, in this study, the accuracy of the theoretical values was improved using the plate vibration model in conjunction with the Miki model. The experimental results showed that when the vibration of the nonwoven sheet was suppressed, the sound absorption coefficient was higher than that of the vibration-prone nonwoven sheet alone. The sound absorption coefficient at the peak frequency was increased by >0.2, especially for 3501BD. Using the support frame, the sound absorption coefficient at the peak frequencies of 3A01A and 3701B was increased to 0.99. In the theoretical analysis of a large-area, vibration-prone nonwoven fabric, in which the vibration of the nonwoven fabric was taken into account, the theoretical values were in agreement with the experimental values, and the accuracy of the theoretical values was improved. Comparing the theoretical values for nonwoven fabrics without high ventilation resistance, the sound absorption coefficient was greater when vibration was not considered. Therefore, it was suggested that the vibration of the nonwoven fabric hinders sound absorption.

## 1. Introduction

The acoustic properties of nonwovens vary along with changes in the fiber diameter, density, ventilation resistance, etc. [1,2,3]. It is also known that thin nonwoven fabric sheets exhibit broad sound absorption curves corresponding to the thickness of the air space behind them. An accurate prediction of the acoustic properties of such sound-absorbing mechanisms is useful in noise engineering applications.

The acoustic properties of nanofiber nonwoven fabrics have recently been studied [4,5], and nonwoven fabrics have attracted attention as sound-absorbing materials. In addition, the acoustic properties of porous materials, such as nonwoven fibers, and sound-absorbing materials, such as permeable membranes, have been studied [6,7,8]. Furthermore, it was observed that sound wave vibration affects the acoustic properties of these thin membranes or plates [9,10,11].

The purpose of this study was to improve the accuracy of the theoretical analysis of sound absorption mechanisms when a back air space is used in nonwoven fabrics. In the case of a nonwoven sheet with a back air space, it can be shown that there is a difference between the experimental results and theoretical analysis results obtained using the Miki model [2] when the area of the nonwoven sheet is large. Therefore, the accuracy of the theoretical values was improved using the plate vibration model [12] in conjunction with the Miki model [2]. The aim was to keep the difference between the experimental and theoretical values of the sound absorption coefficient within 0.1.

In these experiments, the sound absorption coefficient was measured under the following conditions: (1) a sample with a nonwoven fabric sheet and a back air space and (2) a sample with a vibration-suppressed nonwoven fabric sheet and a back air space. A support frame with honeycomb-shaped openings was utilized to suppress the vibration of the nonwoven fabric sheet so as to experimentally reproduce a theoretical model that does not account for vibration.

Experiments were conducted using nonwoven fabrics with different ventilation resistance. Theoretical analysis of the presence or absence of vibration of the nonwoven fabrics was then performed for each of these experimental values.

## 2. Measurement Equipment and Samples

### 2.1. Measurement Equipment

**Figure 1:**
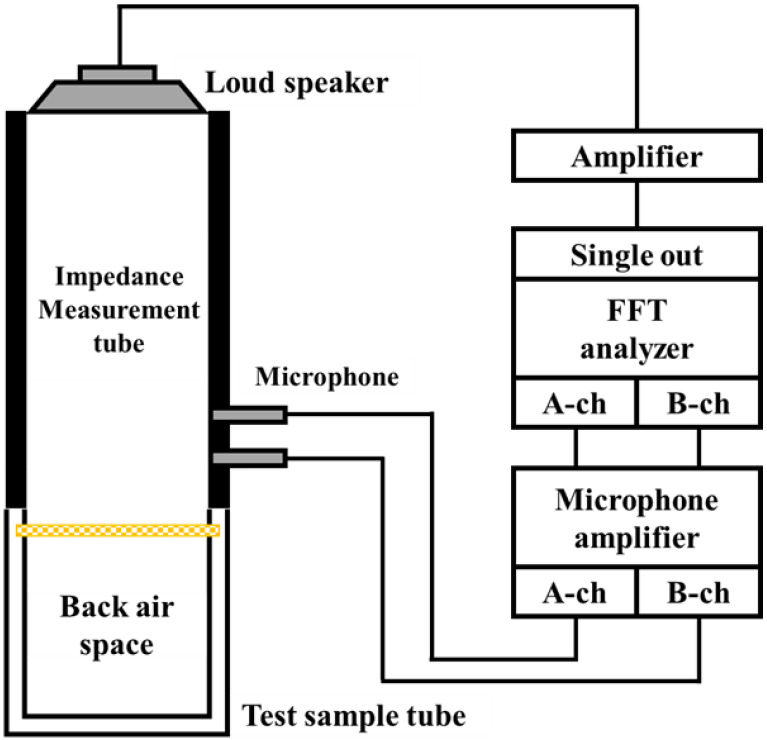
Schematic diagram of a two-microphone impedance tube for absorption coefficient measurements.

Sound absorption coefficients were measured using a Brüel & Kjær Type 4206 two-microphone impedance measuring tube, as shown in the schematic diagram in Figure 1. A sample was attached to the impedance measurement tube, a sinusoidal signal was generated by the signal generator in the fast Fourier transform (FFT) analyzer, and sound waves radiated into the tube by means of a loudspeaker. The FFT analyzer then measured the transfer function between the sound pressure signals from the two microphones attached to the measurement tube. The measured transfer function was used to calculate the normal incident sound absorption coefficient in accordance with ISO 10534-2. The frequency range over which the measurement device can be used is 50–1600 Hz.

The ventilation resistance *R_n_* of nonwoven fabrics was measured using a Kato Tech KES-F8-AP1 permeability tester. This is an air permeability tester that injects a constant flow of air into a sample via a plunger and cylinder. A precise pressure gage was used to quantify the pressure loss caused by the sample at a constant flow rate of 4 cc/cm^2^/s (4 × 10^−2^ m/s). The measurement result allowed the ventilation resistance *R_n_* (kPa s/m) to be calculated directly. Flow resistivity *σ_n_* was calculated by dividing the ventilation resistance *R_n_* by the thickness of the nonwoven fabric.

### 2.2. Measurement Samples

**Figure 2:**
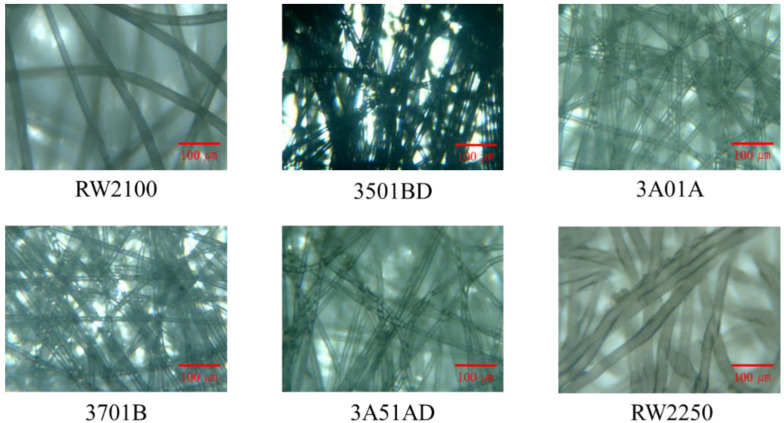
Microphotographs of nonwoven fabric sheets.

Figure 2 shows microphotographs of the six nonwoven fabric sheet samples used in this study. The nonwoven fabric sheet samples were cut into a circular shape with a diameter of 110 mm. The nonwoven fabrics used in the experiment were spun-bound fabrics, which were manufactured in thin sheets with fibers that were almost perpendicular to the thickness direction.

Table 1 shows the specifications of each nonwoven fabric with respect to ventilation resistance. These nonwovens differed in terms of their ventilation resistance, thickness, and area density, where flow resistivity *σ_n_* is the ventilation resistance *R_n_* divided by the thickness *t_n_* of the nonwoven sheet.

**Figure 3:**
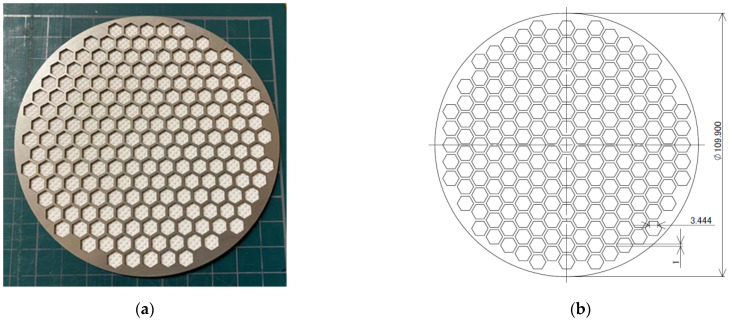
Frame for supporting the nonwoven fabric: (**a**) photograph; (**b**) dimensions of the frame.

Figure 3a,b show a photograph of a nonwoven fabric with a support frame and a schematic diagram of the support frame, respectively. The nonwoven fabric with a support frame shown is a sample in which the vibration of the nonwoven fabric was suppressed by pasting the nonwoven fabric onto a support frame, which was made out of SUS304. The outer diameter *D_f_* = 109.90 mm, thickness *t_f_* = 1.00 mm, the width of the frame was 1.0 mm, and aperture was a regular hexagonal honeycomb shape, where each face was 3.44 mm long. The aperture ratio was as large as 0.73 to ensure that the mesh was acoustically negligible.

**Figure 4:**
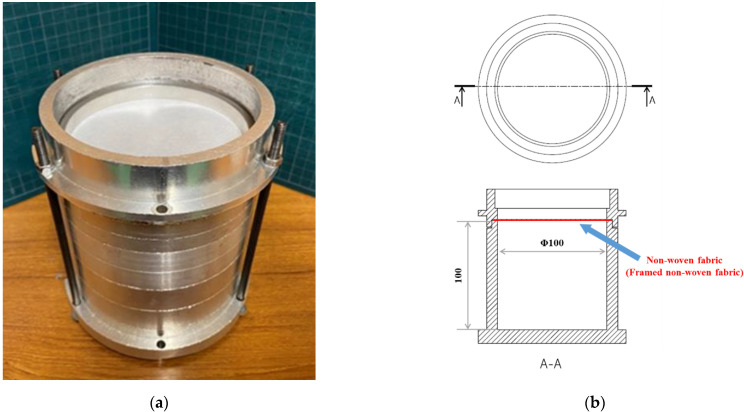
Sample tube: (**a**) photograph; (**b**) dimensions.

Figure 4a,b present a photograph and schematic diagrams of the sample tubes, respectively. The material of the sample tube was aluminum alloy, and the dimensions were: inner diameter *D_i_* = 100 mm, outer diameter 120 mm, and length of the back air space *L* = 100 mm.

As shown in Figure 4b, a sample was placed at the top end of the sample tube, and the sound absorption coefficient was measured for a nonwoven fabric sheet with a back air space. In this paper, the vibration of the nonwoven fabric was created via self-excited vibration caused by sound waves incident on the nonwoven fabric. In this experiment, the vibration of the nonwoven fabric was excited via the sound wave in order to measure the sound absorption coefficient.

## 3. Theoretical Analyses

### 3.1. Analysis Model Corresponding to the Measured Sample

In this section, the sound absorption coefficient of nonwoven fabric with a back air space was derived through theoretical analysis using a transfer matrix.

**Figure 5:**
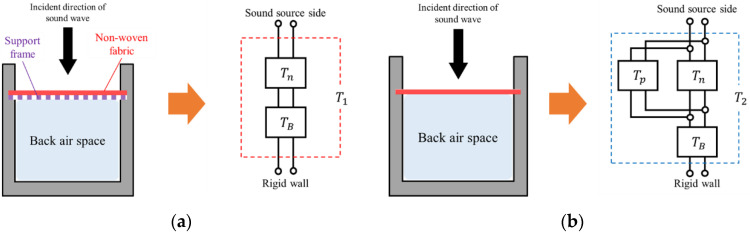
Equivalent circuits corresponding to test samples: (**a**) framed nonwoven fabric; (**b**) nonwoven fabric sheet.

Figure 5a,b show the equivalent circuits corresponding to the measured samples. Figure 5a is a schematic of a nonwoven fabric sample with a support frame, and Figure 5b is a schematic of a nonwoven sheet sample. The right-hand sides of Figure 5a,b show equivalent circuits corresponding to the analytical model described in Section 3.6, respectively.

First, the analytical model shown in Figure 5a did not consider the vibration of the nonwoven fabric because the vibration of the nonwoven fabric was suppressed by the support frame. However, the analytical model in Figure 5b considered the vibration of the nonwoven fabric. Therefore, it can be seen that there were three types of elements that composed these analytical models: the porous characteristics of the nonwoven fabric, the air layer behind the fabric, and the vibration of the nonwoven fabric.

The mathematical expressions of the equivalent circuits on the right side of Figure 5a,b are explained in Section 3.6.

### 3.2. Transfer Matrix Based on a One-Dimensional Wave Equation

Assuming that the sound pressure and particle velocity at the incident surface of the sound wave are *p*_1_ and *u*_1_, respectively, the sound pressure and particle velocity at the end of the measurement tube are *p*_2_ and *u*_2_, respectively, and the cross-sectional area of the measurement tube is *S*. The transfer matrix *T* between the incident and end surfaces can be expressed using the one-dimensional wave equation as follows [13]:

(1) $$\left[\begin{array}{c} p_{1}\\ {\mathrm{Su}}_{1} \end{array}\right]=T\left[\begin{array}{c} p_{2}\\ {\mathrm{Su}}_{2} \end{array}\right]=\left[\begin{array}{cc} A & B\\ C & D \end{array}\right]\left[\begin{array}{c} p_{2}\\ {\mathrm{Su}}_{2} \end{array}\right]$$

where the four-terminal constants *A*, *B*, *C*, and *D* in the transfer matrix *T* are expressed as follows:

(2) $$T=\left[\begin{array}{cc} A & B\\ C & D \end{array}\right]=\left[\begin{array}{cc} \cos h(\gamma l) & \frac{Z_{c}}{S}\sin h(\gamma l)\\ \frac{S}{Z_{c}}\sin h(\gamma l) & \cos h(\gamma l) \end{array}\right]$$

where *γ* is the propagation constant, *Z_c_* is the characteristic impedance, and *l* is the length of the measurement tube. When Equation (2) is substituted for *A*, *B*, *C*, and *D* in Equation (1) and expressed as a series of equations for *p*_1_ and *u*_1_, they are one-dimensional wave equations for sound pressure and particle velocity.

### 3.3. Transfer Matrix for Nonwoven Fabrics as Porous Materials

The propagation constant *γ_n_* and characteristic impedance *Z_n_* for a nonwoven fabric can be expressed as in Equations (3) and (4) using the Miki model [2]. Note that *j* is an imaginary unit.

(3) $$\gamma_{n}=\rho c\left\{\;1+0.0699{\left(\frac{f}{\sigma_{n}}\right)}^{-0.632}\right\}-\;j\rho c\left\{0.107{\left(\frac{f}{\sigma_{n}}\right)}^{-0.632}\right\}\;$$

(4) $$Z_{n}=0.160\frac{\omega }{c}{\left(\frac{f}{\sigma_{n}}\right)}^{-0.618}+j\frac{\omega }{c}\left\{1+0.109{\left(\frac{f}{\sigma_{n}}\right)}^{-0.618}\right\}\;$$

The flow resistivity *σ_n_* of the nonwoven fabric in Equations (3) and (4) can be expressed as Equation (5).

(5) $$\sigma_{n}=\frac{R_{n}}{t_{n}}\;$$

where *R_n_* is the ventilation resistance of the nonwoven fabric and *t_n_* is the thickness of the nonwoven fabric.

The transfer matrix *T_n_* of the nonwoven fabric is obtained by substituting Equations (3) and (4) into the transfer matrix in Equation (2) as follows to yield:

(6) $$T_{n}=\left[\begin{array}{cc} \cos h\left(\gamma_{n}t_{n}\right) & \frac{Z_{n}}{S_{n}}\sin h\left(\gamma_{n}t_{n}\right)\\ \frac{S_{n}}{Z_{n}}\sin h\left(\gamma_{n}t_{n}\right) & \cos h\left(\gamma_{n}t_{n}\right) \end{array}\right]=\left[\begin{array}{cc} A_{n} & B_{n}\\ C_{n} & D_{n} \end{array}\right]\;$$

### 3.4. Transfer Matrix for the Vibration of Nonwoven Sheet

The acoustic impedance Z*_p_* for a vibrating sheet is expressed using Equation (7) [12].

(7) $$Z_{p}=\frac{b}{S_{n}^{2}}+j\frac{\omega m}{S_{n}^{2}}=\frac{2\xi \sqrt{{\mathrm{mk}}_{a}}}{S_{n}^{2}}+j\frac{{\omega \rho }_{A}S_{n}}{S_{n}^{2}}=\frac{{2\xi k}_{a}}{\omega_{0}S_{n}^{2}}+j\frac{{\omega \rho }_{A}}{S_{n}}\;$$

where *S_n_* is the area of the nonwoven fabric, *ρ_A_* is the area density of the nonwoven fabric, *ξ* is the damping ratio, and *ω* is the angular frequency of the sound wave (*ω* = 2π*f*, *f*: frequency). The attenuation constant *b* of the nonwoven fabric, mass *m* of the nonwoven fabric, natural angular frequency *ω*_0_, and spring constant *k_a_* of the air layer can be expressed using Equations (8)–(11), respectively, as follows

(8) $$b=2\xi \sqrt{{\mathrm{mk}}_{a}}\;$$

(9) $${m=\rho }_{A}S_{n}\;$$

(10) $$\omega_{0}=\sqrt{\frac{k_{a}}{m}}\;$$

(11) $$k_{a}=\frac{{\Gamma p}_{0}S_{t}}{L}\;$$

where *Γ* is the specific heat ratio, *p*_0_ is the atmospheric pressure, *S_t_* is the cross-sectional area of the measurement tube (*S_t_* = *S_n_*), and *L* is the length of the back air layer. The damping ratio *ξ* is set to 0.1.

Substituting Equation (7) for *Z_p_* in the following equation, the transfer matrix *T_p_* of the vibrating sheet is expressed using the right side of the following equation:

(12) $$T_{p}=\left[\begin{array}{cc} 1 & Z_{p}\\ 0 & 1 \end{array}\right]=\left[\begin{array}{cc} 1 & \frac{{2\xi k}_{a}}{\omega_{0}S_{n}^{2}}+j\frac{{\omega \rho }_{A}}{S_{n}}\\ 0 & 1 \end{array}\right]=\left[\begin{array}{cc} A_{p} & B_{p}\\ C_{p} & D_{p} \end{array}\right]\;$$

### 3.5. Transfer Matrix for Back Air Space

The transfer matrix *T_B_* for the back air space can be expressed as follows, assuming that damping in the transfer matrix based on the one-dimensional wave equation in Equation (2) is negligible:

(13) $$T_{\;B}=\left[\begin{array}{cc} \cos\mathrm{kL} & j\frac{\rho c}{S_{t}}\sin\mathrm{kL}\\ j\frac{S_{t}}{\rho c}\sin\mathrm{kL} & \cos\mathrm{kL} \end{array}\right]\;$$

where *k* is the wavenumber, *ρ* is the density of air, and *c* is the speed of sound in air.

### 3.6. Equivalent Circuit and Transfer Matrix Corresponding to the Analytical Model

For the nonwoven fabric with a support frame in Figure 5a, the transfer matrix *T*_1_ can be obtained via the cascade connecting the transfer matrix *T_n_* using the Miki model in Equation (6) and the transfer matrix *T_B_* for the back air space in Equation (13), as follows:

(14) $$\begin{array}{cc} {\;T}_{1} & =T_{n}\times T_{B}\\ & =\left[\begin{array}{cc} \cos h\left(\gamma_{n}t_{n}\right) & \frac{Z_{n}}{S_{n}}\sin h\left(\gamma_{n}t_{n}\right)\\ \frac{S_{n}}{Z_{n}}\sin h\left(\gamma_{n}t_{n}\right) & \cos h\left(\gamma_{n}t_{n}\right) \end{array}\right]\times \left[\begin{array}{cc} \cos\mathrm{kL} & j\frac{\rho c}{S_{t}}\sin\mathrm{kL}\\ j\frac{S_{t}}{\rho c}\sin\mathrm{kL} & \cos\mathrm{kL} \end{array}\right]\\ & =\left[\begin{array}{cc} A_{1} & B_{1}\\ C_{1} & D_{1} \end{array}\right] \end{array}$$

In addition, when the acoustic elements are connected sequentially, such as in silencer design, the transfer matrix cascades [13].

For the nonwoven sheet shown in Figure 5b alone, the transfer matrix *T_n_* using the Miki model in Equation (6) and the transfer matrix *T_p_* for the vibration of the nonwoven sheet in Equation (12) are connected in parallel [12,14,15]. The parallel connection occurs because there are two sound-absorbing principles for one incident surface [12,14]. Owing to the parallel connection, sound pressure acts equally on both sound-absorbing principles and particle velocity is diverted more toward the sound-absorbing principle with lower impedance. Next, the transfer matrix *T*_2_ is obtained via a cascade connecting the transfer matrix *T_B_* of the back air layer in Equation (13) to them, as shown in the following Equation [13].

(15) $$\begin{array}{cc} T_{2} & =\left[\begin{array}{cc} \frac{A_{n}B_{p}{+A}_{p}B_{n}}{B_{n}{+B}_{p}} & \frac{B_{n}B_{p}}{B_{n}{+B}_{p}}\\ C_{n}{+C}_{p}+\frac{\left(A_{p}-A_{n}\right)\left(D_{n}-D_{p}\right)}{B_{n}{+B}_{p}} & \frac{D_{n}B_{p}{+D}_{p}B_{n}}{B_{n}{+B}_{p}} \end{array}\right]\left[\begin{array}{cc} \cos\mathrm{kL} & j\frac{\rho c}{S_{t}}\sin\mathrm{kL}\\ j\frac{S_{t}}{\rho c}\sin\mathrm{kL} & \cos\mathrm{kL} \end{array}\right]\\ & \begin{array}{c} =\left[\begin{array}{cc} A_{2} & B_{2}\\ C_{2} & D_{2} \end{array}\right] \end{array} \end{array}$$

### 3.7. Derivation of the Sound Absorption Coefficient

The sound absorption coefficient for the transfer matrices *T*_1_ and *T*_2_ obtained in Section 3.6 was calculated. The four-terminal constants of the transfer matrices *T*_1_ and *T*_2_ correspond to the four-terminal constants *A*, *B*, *C*, and *D* of the transfer matrix *T* in Equation (1).

Since the ends of *T*_1_ and *T*_2_ are rigid walls, *u*_2_ = 0; therefore, Equation (1) can be expressed as:

(16) $$\left[\begin{array}{c} p_{1}\\ {\mathrm{Su}}_{1} \end{array}\right]=\left[\begin{array}{c} {\mathrm{Ap}}_{2}\\ {\mathrm{Cp}}_{2} \end{array}\right]\;$$

The specific acoustic impedance *Z* of this acoustic system, as seen from the plane of incidence, is shown in Equation (17):

(17) $$Z=\frac{p}{u}$$

where, from *p* = *p*_1_ and *Su*_1_ = *S_t_u*, the specific acoustic impedance *Z* is expressed as in Equation (18).

(18) $$Z=\frac{p}{u}=\frac{p}{S_{t}u}S_{t}=\frac{p_{1}}{{\mathrm{Su}}_{1}}S_{t}=\frac{A}{C}S_{t}\;$$

Here, the relationship between specific acoustic impedance *Z* and reflectance *R* is expressed using the following equation

(19) $$R=\frac{Z-\rho c}{Z+\rho c}$$

The sound absorption coefficient *α* is expressed using the reflection coefficient *R* as follows

(20) $$\alpha =1-{\left|R\right|}^{2}$$

## 4. Comparisons of Experimental and Theoretical Values

**Figure 6:**
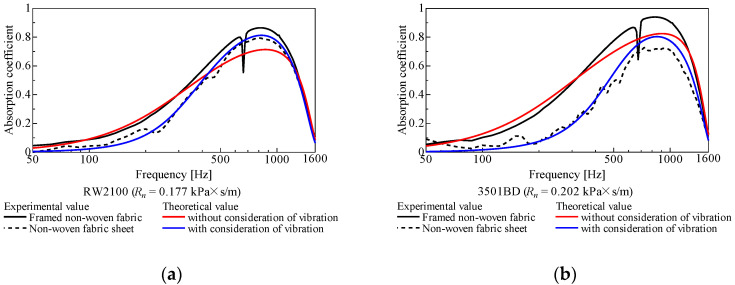

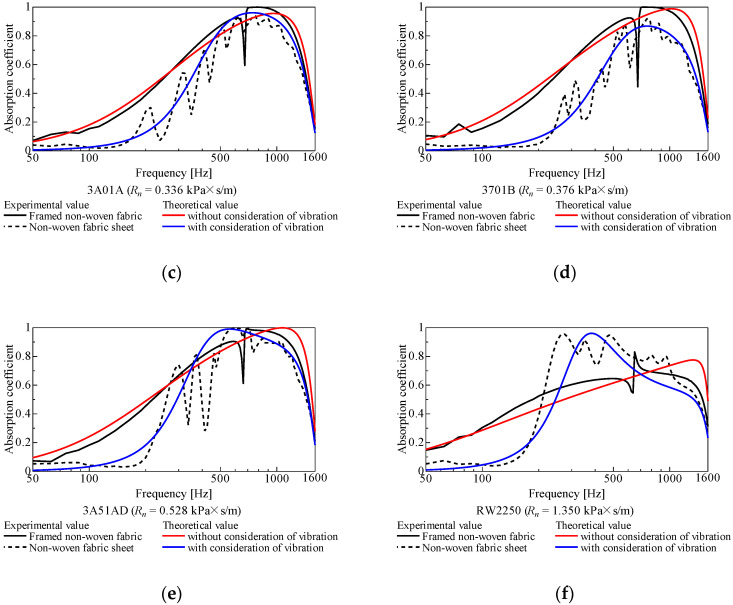
Comparison of experimental and theoretical values (length of back air space L = 100 mm): (**a**) RW2100; (**b**) 3501BD; (**c**) 3A01A; (**d**) 3701B; (**e**) 3A51AD; (**f**) RW2250.

A comparison between the sound absorption coefficient based on the theoretical analysis and the experimental results is shown in Figure 6a–f.

In Figure 6a–d, for both nonwoven sheets, the experimental values for the nonwoven fabric with a support frame (black line) were consistent with the theoretical values (red line), which did not account for the vibration of the nonwoven fabric. In Figure 6e,f, the trends of the experimental (black line) and theoretical (red line) values were in agreement at low frequencies, but less so at high frequencies.

In Figure 6a–d, the experimental values for the nonwoven sheet alone (black dashed line) were consistent with the theoretical values (blue line), which considered the vibration of the nonwoven fabric. Similarly, in the case of the nonwoven sheet alone, the agreement between the experimental (black dashed line) and theoretical (blue line) values was poor for Figure 6e,f, but much better in the low-frequency range.

As described above, the relatively good agreement between the experimental and theoretical trends suggests that the vibration of the nonwoven fabric with the support frame was nearly suppressed, and that of the nonwovens alone suggested that the nonwovens were vibrating. This is because the support frame reduced the mass of the nonwoven fabric per aperture to less than 1/100 and the frequency band in which the nonwoven fabric’s spring-mass system vibrates is considered to be approximately one order of magnitude higher. Therefore, the vibration of the nonwoven fabric in the frequency band of interest is considered to be nearly completely suppressed in terms of the sound absorption coefficient.

Meanwhile, the vibration of the support frame itself, independently of the type of nonwoven fabric, was observed as a spike-like decrease in sound absorption coefficient near 650 Hz.

The effect of the vibration component in the analytical model is also discussed. In the theoretical values shown in Figure 6a–e, the sound absorption coefficient is generally higher over the entire frequency range when vibration is not considered. The simulation results indicate that the vibration of the nonwoven fabric hindered sound absorption for nonwoven fabrics that do not have high ventilation resistance.

For RW2100, shown in Figure 6a, the difference in experimental values with and without the support frame was the smallest among the nonwovens used in this study. The theoretical values also showed the smallest difference depending on whether vibration was taken into account. This suggests that when the ventilation resistance was low, as in the case of RW2100, the excitation force due to sound pressure was low, and thus the nonwoven fabric was less likely to vibrate.

RW2250, shown in Figure 6f, was the only nonwoven fabric in this study to show a higher sound absorption coefficient experimentally with a single nonwoven fabric than with a support frame (except in the low-frequency range). Additionally, the theoretical sound absorption coefficient was higher when vibration was taken into account than when it was not. This suggests that RW2250 had a significantly higher ventilation resistance than the other nonwovens used in this study, and its effectiveness as a plate vibration type sound absorber was better than its functionality as a porous (permeable) material, and thus it showed a different trend compared to the other nonwovens sheets.

## 5. Conclusions

The following conclusions were obtained from the experimental results and the theoretical analysis of a nonwoven fabric sheet with a back air space.

The vibration of the nonwoven fabric was suppressed by attaching a support frame with honeycomb-shaped apertures to the large-area, vibration-prone nonwoven fabric. This led to an experiment corresponding to a theoretical analytical model that did not account for the vibration of the nonwoven fabric. The experimental results showed that when the vibration of the nonwoven sheet was suppressed, the sound absorption coefficient was higher than that of the vibration-prone nonwoven sheet alone. The sound absorption coefficient at the peak frequency was increased by >0.2, especially for 3501BD. Using the support frame, the sound absorption coefficient at the peak frequencies of 3A01A and 3701B was increased to 0.99. However, the opposite trend was observed for RW2250, which showed the highest ventilation resistance.

In the theoretical analysis of a large-area, vibration-prone nonwoven fabric, in which the vibration of the nonwoven fabric was taken into account, the theoretical values agreed with the experimental values, and the accuracy of the theoretical values was improved.

By comparing the theoretical values for nonwoven fabrics without high ventilation resistance, the sound absorption coefficient was greater when vibration was not considered. Therefore, the results suggested that the vibration of the nonwoven fabric hindered sound absorption.

The following secondary points were also found: A comparison of the theoretical values for nonwoven fabrics with high ventilation resistance showed that the sound absorption coefficient was higher when vibration was considered. However, this was not the case in the low-frequency range.

The trends observed for the theoretical values were generally consistent with those of the experimental values.

## Figures and Tables

**Table 1:** Specifications of nonwoven fabrics.

Name	Ventilation Resistance $R_{n}\;(\mathrm{kPa}\;\times \;s/m)$	Thickness $t_{n}\;(\mathrm{mm})$	Flow Resistivity $\sigma_{n}{=R}_{n}/t_{n}\;(\mathrm{kPa}\;\times \;s/m^{2})$	Area Density $\rho_{A}\;(g/m^{2})$	Material
RW2100	0.1767	0.58	303.6	100	Polypropylene
3501BD	0.2021	0.19	1064	50	Polyester
3A01A	0.3363	0.39	862.2	100	Polyester
3701B	0.3763	0.24	1568	70	Polyester
3A51AD	0.5283	0.45	1174	152	Polyester
RW2250	1.350	0.82	1646	250	Polypropylene
